# Profiles of immune infiltration in abdominal aortic aneurysm and their associated marker genes: a gene expression-based study

**DOI:** 10.1590/1414-431X2021e11372

**Published:** 2021-09-03

**Authors:** Tan Li, Tianlong Wang, Xin Zhao

**Affiliations:** 1 Department of Cardiovascular Ultrasound, the First Hospital of China Medical University, Shenyang, China; 2 The First Clinical College of China Medical University, the First Hospital of China Medical University, Shenyang, China; 3 Department of Operation Room, the First Hospital of China Medical University, Shenyang, China

**Keywords:** Abdominal aortic aneurysm, Immune infiltration, Immune microenvironment, Genomics, Bioinformatics

## Abstract

Immune-mediated inflammation plays a key role in the pathology of abdominal aortic aneurysm (AAA). We aimed to use a computational approach to profile the immune infiltration patterns and related core genes in AAA samples based on the overexpression of gene signatures. The microarray datasets of AAA and normal abdominal tissues were acquired from gene expression omnibus (GEO) database. We evaluated the composition of immune infiltrates through microenvironment cell populations (MCP)-counter. Weighted gene correlation network analysis (WGCNA) was employed to construct the co-expression network and extract gene information in the most relevant module. Functional and pathway enrichment analysis was performed and immune infiltration related core genes were screened. AAA tissues had a higher level of infiltration by cytotoxic lymphocytes, NK cells, T cells, fibroblasts, myeloid dendritic cells, and neutrophils than normal aorta. The red module was strongly correlated with the infiltrating levels of T cells and cytotoxic lymphocytes. Gene ontology (GO) and pathway analyses revealed that genes in the most relevant module were mainly enriched in T cell activation, regulation of lymphocyte activation, cytokine-cytokine receptor interaction, and chemokine signaling pathway, etc. The expression of GZMK, CCL5, GZMA, CD2, and EOMES showed significant correlations with cytotoxic lymphocytes, while CD247, CD2, CD6, RASGRP1, and CD48 expression were positively associated with T cell infiltration. In conclusion, we comprehensively analyzed profiles of infiltrated immune cells in AAA tissues and their associated marker genes. Our data may provide a novel clue to indicate the underlying molecular mechanisms of AAA formation in terms of immune infiltration.

## Introduction

Abdominal aortic aneurysm (AAA) is a complicated and multifactorial disease, which represents a pathological expansion of the abdominal aorta with a diameter of ≥3.0 cm or more than 50% of normal diameter. AAA is considered one of the leading causes of mortality in subjects aged over 65 years worldwide (1). Although there are several identified biological features in AAA, compelling evidence suggests that immune-mediated processes play a prominent and defining role in the pathogenesis of AAA (2,3). The immune-inflammatory responses are mediated by a number of specialized immune cell types that interplay in a highly coordinated manner and are functionally critical to AAA initiation and progression (4).

Based on previous human and experimental AAA studies, several exogenous immune cells, including lymphocytes, macrophages, neutrophils, natural killer (NK) cells, and dendritic cells, have been found to infiltrate into the aneurysmal tissues, evoking a series of inflammatory reactions by releasing a wide range of pro-inflammatory cytokines that contribute to the direct structural protein degradation of the abdominal aorta (3,5,6). However, most reports just focused on a narrow view of immune response, generally discussing only one or two cell types using immunohistochemistry (IHC), immunofluorescence, or flow cytometry. Little is known about the composition and diversity of infiltrative immune cells that collectively influence the risk of AAA. To better understand the complex network of immune cells acting in AAA, it is necessary to simultaneously quantify multiple immune-related infiltrates within a tissue specimen for determining their specialized roles in AAA pathophysiology.

Advances in computational methods have reinvigorated the potential for large public repositories for the collections of genomic data, which serves to offer more comprehensive information about complex diseases like AAA (7). Large-scale programs have allowed for the identification of immune microenvironment and even the performance of immune strategies in AAA, which increasingly attracts researchers' interests. The microenvironment cell populations (MCP)-counter method measures the inter-sample relative abundance of different cell groups in a microenvironment and across simulated mixtures (8,9). This method can simultaneously quantify ten cell types with a single gene-expression assay. Compared to CIBERSORT algorithm, there is a conceptual difference. CIBERSORT is a flow cytometry-inspired computational method and commonly used to estimate the intra-sample proportions of immune cell subtypes within the leukocyte fraction of simulated mixtures (8). So far, CIBERSORT algorithm has been employed to evaluate the infiltration of immune cells in AAA samples (10). Although MCP-counter analysis is widely conducted in studies of tumor microenvironment (11-14), its realistic application in AAA has not been validated.

Therefore, the present study mainly applied MCP algorithm to calculate the infiltrating abundance of immune cells in AAA samples. Then, gene co-expression network was constructed to investigate the gene modules and their associations with immune infiltrates in AAA, and we further carried out functional annotation of genes in the most relevant module and screened for immune infiltration-related core genes. Our findings provided some significant insights into the complex association between immune microenvironment and AAA formation.

## Material and Methods

### Data acquisition

The gene expression omnibus (GEO) database is a public database of gene chip data. We searched for the available datasets related to AAA in the GEO database. Three chips (GSE47472, GSE57691, and GSE98278) related to AAA were annotated with Illumina HumanHT-12 v4.0 expression bead chip (www.illumina.com). Relevant data were extracted from the three chips, in which 8 normal cases were extracted from GSE47472, 49 AAA and 10 normal cases were extracted from GSE57691, and 48 AAA cases were extracted from GSE98278. Due to the batch effect between different data, the original datasets underwent background correction and quantile normalization by “Limma” package (https://bioconductor.org).

### Evaluation of AAA-relevant immune infiltration

The MCP-counter package in R (https://github.com) was applied to evaluate the immune infiltration of each tissue sample from transcriptomic data. This method quantifies the abundance of different immune cells on the basis of specific molecular markers (8). Then, we appraised the association degree between different infiltrates in AAA and explored AAA-specific immune infiltrating components. xCELL algorithm (https://xcell.ucsf.edu) was also utilized to determine the abundance of each cell component within tissues between AAA and normal aorta.

### AAA-related gene co-expression network construction by WGCNA analysis

Due to the noise in the second-generation sequencing technology, median absolute deviation (MAD) approach was employed to reduce the presence of noise. Gene co-expression network was constructed by the weighted gene correlation network analysis (WGCNA) R software package (https://horvath.genetics.ucla.edu). In the WGCNA technique, we used power value to calculate the co-expression module among genes. The criterion of co-expression weight >2.5 was used to select the candidate network. We calculated the correlation between modules and AAA risk-related immune infiltrating components. Heatmap was applied to describe the strength of relationship (strong or weak). Then, we selected the most relevant module for the next analysis.

### Functional and pathway enrichment analysis of genes in the most relevant module

Gene ontology (GO) analysis (http://geneontology.org) is a major bioinformatics tool for annotating gene and its products. It contains terms for three categories: cellular components, molecular functions, and biological processes. The Kyoto encyclopedia of genes and genomes (KEGG) is a database (https://www.genome.jp) with information on genomes, biological pathways, diseases, and chemicals. To investigate the potential biological themes and pathways of genes in the most relevant module, we used the clusterProfiler package in R (https://bioconductor.org) for GO and KEGG analyses.

### Immune infiltration-related core genes

Correlation analysis was conducted to determine the association between genes in the most relevant module and components of immune infiltration. The top 5 correlated genes were searched as core genes affecting the immune infiltration of AAA.

### Statistical analysis

All analyses were conducted with R version 3.5 (https://www.r-project.org/) and its several open packages. Differences between groups of immune infiltrating components were examined by nonparametric tests. The detection of the core genes of immune infiltration was assessed by Spearman's correlation. For all the multiple tests, we utilized Benjamini and Hochberg (BH) to correct the P value. The two-sided P value <0.05 was considered statistically significant. Other used visual packages included pheatmap, ggplot2, and corrplot.

## Results

### Landscape of immune infiltration in AAA tissues

**Figure 1:**
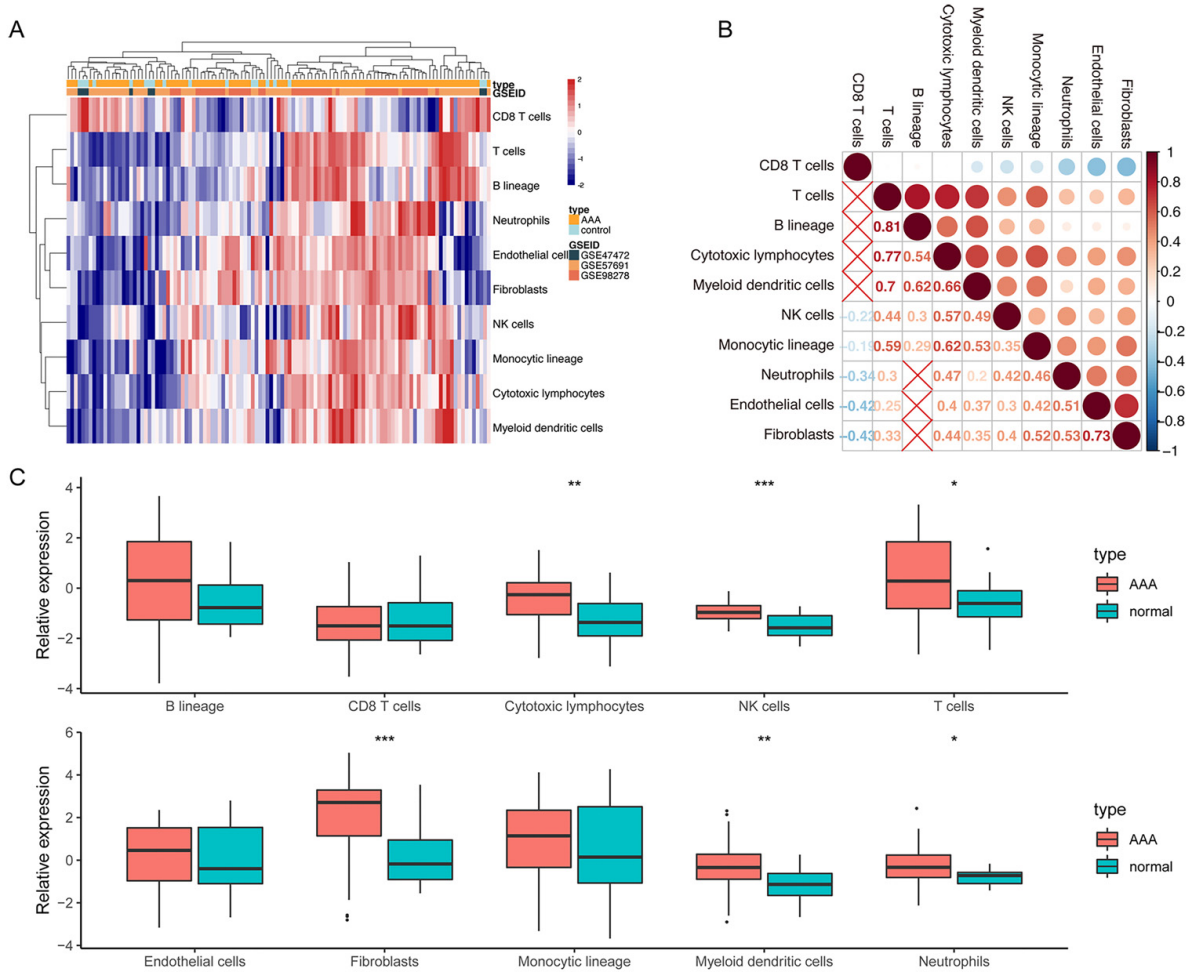
The landscape of immune infiltration in abdominal aortic aneurysm (AAA) samples. **A**, The performance of microenvironment cell populations (MCP)-counter for characterizing immune infiltration in AAA and normal aortic tissues. **B**, Correlation analysis between cell components with significant results. **C**, The differences of immune infiltration between AAA and normal aortic tissues. Data are reported as median and interquartile range. *P<0.05, **P<0.01, ***P<0.001 (Mann-Whitney U test).

Eight immune (T cells, CD8 T cells, NK cells, cytotoxic lymphocytes, B lineage, monocytic lineage, myeloid dendritic cells, and neutrophils) and two stromal (endothelial cells and fibroblasts) cell populations in AAA and normal aortic tissues were discriminated by MCP-counter method (Figure 1A). Through correlation analysis, we found a relationship between different components in AAA (Figure 1B). In particular, there was a strong positive association of T cells with B lineage and cytotoxic lymphocytes.

Furthermore, we confirmed the differences of immune infiltrating components between AAA and normal tissues. Results indicated that AAA tissues contained a higher proportion of T cells, cytotoxic lymphocytes, NK cells, fibroblasts, myeloid dendritic cells, and neutrophils compared with normal tissues (all P<0.05) (Figure 1C). In addition, based on xCELL analysis, we screened out twelve cell types including CD4+ T cells with significant differences between AAA and normal aorta (Supplementary Figure S1). However, there were no significant changes in other cell populations such as macrophages, Tregs, and MSC (Supplementary Table S1).

### AAA-related gene co-expression network construction

**Figure 2:**
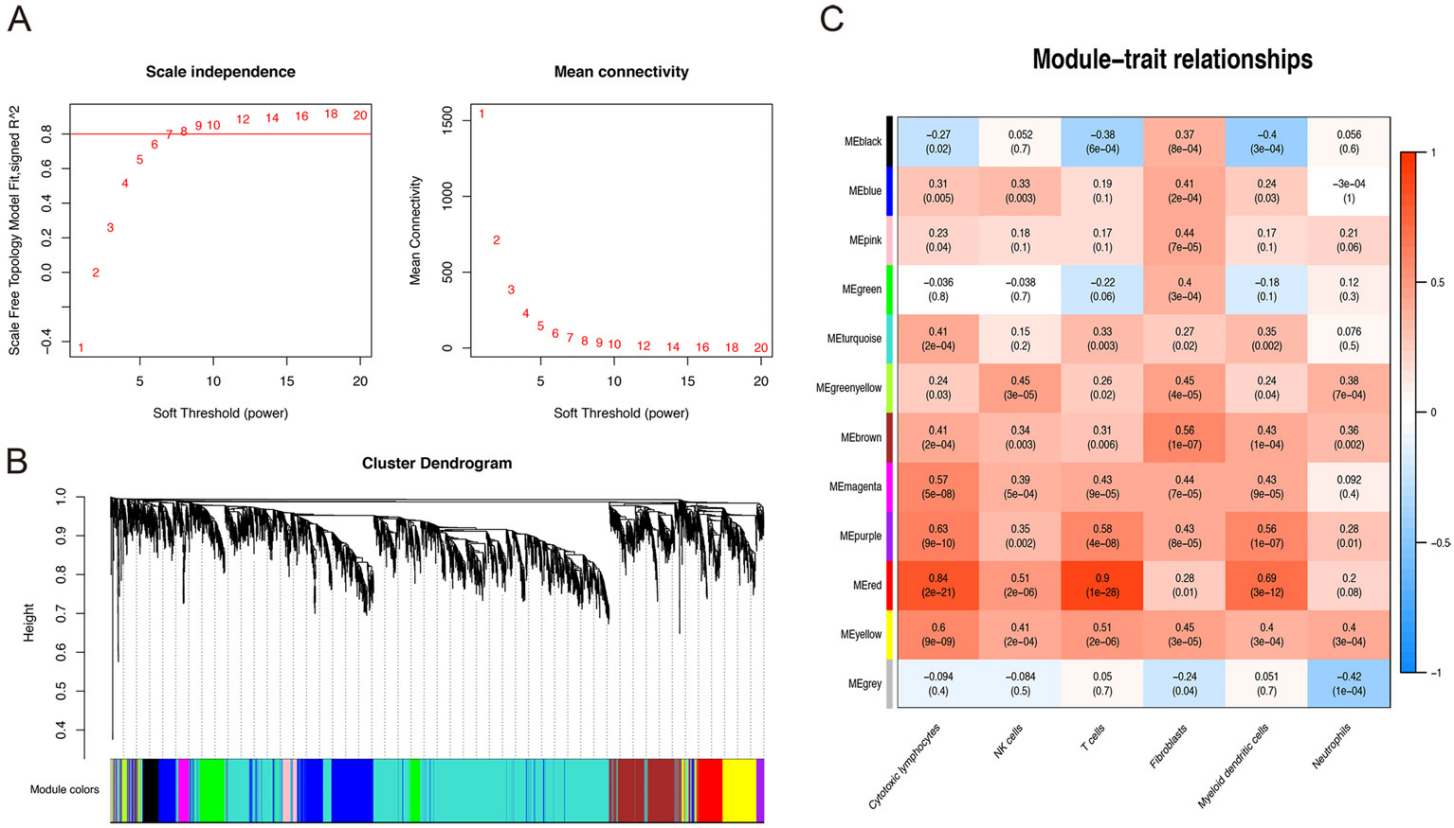
Weighted gene correlation network analysis (WGCNA) analysis for abdominal aortic aneurysm (AAA)-related gene co-expression network construction. **A**, Soft threshold selection. **B**, Gene dendrogram obtained by average linkage hierarchical clustering. Each color represents a gene module. **C**, Correlation analysis of WGCNA modules with AAA-related immune infiltrates. The numbers represent the correlation coefficient (P value) on the horizontal and vertical coordinates.

The WGCNA analysis was used for describing the correlation models among genes across microarray samples. First, the MAD test was carried out on the data and the top 25% expressed genes were selected for the next step analysis. Then, 4726 genes were obtained for further study. Through soft threshold selection, we used soft threshold 7 for module building (Figure 2A). Finally, 11 modules were obtained, and there were 2240, 776, 499, 303, 285, 205, 112, 107, 78, 57, and 37 genes in the modules, respectively (Figure 2B) (Supplementary Table S2). In addition, we analyzed the relationship between different modules and AAA risk-related immune infiltrates. Interestingly, the results suggested that the red module had a strong positive correlation with the infiltrating levels of T cells and cytotoxic lymphocytes (r=0.90 and 0.84 respectively, all P<0.05) (Figure 2C).

### Functional and pathway enrichment analysis of genes in identified module

**Figure 3:**
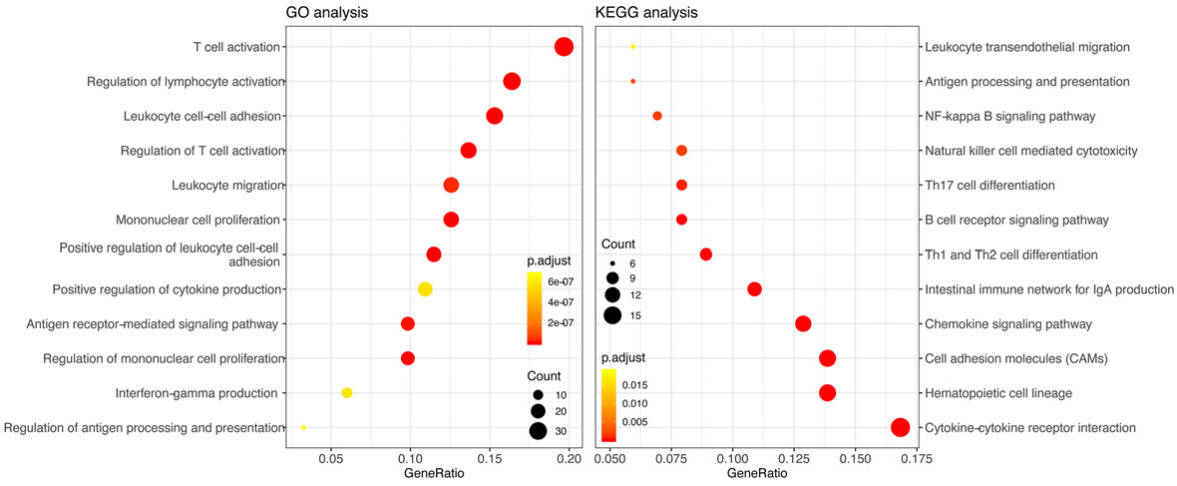
Functional and pathway enrichment analysis of genes in identified module. GO: Gene ontology; KEGG: Kyoto encyclopedia of genes and genomes.

We carried out the functional and pathway enrichment analysis of genes in the identified module. GO analysis revealed that genes in the red module mainly participated in T cell activation, regulation of lymphocyte activation, leukocyte cell-cell adhesion, lymphocyte and mononuclear cell proliferation, etc. Moreover, several pathways linked to AAA were observed trough KEGG analysis, including cytokine-cytokine receptor interaction, chemokine signaling pathway, Th1 and Th2 cell differentiation, and NF-kappa B signaling pathway (Figure 3) (Table 1).

**Table 1:** Functional and pathway enrichment analysis of genes in identified module.

Terms/ID	Description	GeneRatio	P value	P_adjusted_	Count
GO					
GO: 0042110	T cell activation	36/183	1.55E-22	3.62E-19	36
GO: 0007159	Leukocyte cell-cell adhesion	28/183	1.90E-18	2.22E-15	28
GO: 0051249	Regulation of lymphocyte activation	30/183	4.04E-16	2.41E-13	30
GO: 0050863	Regulation of T cell activation	25/183	4.12E-16	2.41E-13	25
GO: 0050870	Positive regulation of T cell activation	21/183	9.22E-16	4.32E-13	21
GO: 0046651	Lymphocyte proliferation	23/183	1.79E-15	7.00E-13	23
GO: 0032943	Mononuclear cell proliferation	23/183	2.12E-15	7.07E-13	23
GO: 1903039	Positive regulation of leukocyte cell-cell adhesion	21/183	2.98E-15	8.71E-13	21
GO: 0022409	Positive regulation of cell-cell adhesion	22/183	6.74E-15	1.75E-12	22
GO: 0070661	Leukocyte proliferation	23/183	8.10E-15	1.89E-12	23
KEGG					
hsa04640	Hematopoietic cell lineage	14/101	1.59E-11	1.57E-09	14
hsa04672	Intestinal immune network for IgA production	11/101	1.80E-11	1.57E-09	11
hsa04514	Cell adhesion molecules (CAMs)	14/101	4.11E-09	2.38E-07	14
hsa04060	Cytokine-cytokine receptor interaction	17/101	1.66E-07	7.23E-06	17
hsa04062	Chemokine signaling pathway	13/101	8.29E-07	1.80E-05	13
hsa04658	Th1 and Th2 cell differentiation	9/101	2.44E-06	3.26E-05	9
hsa04662	B cell receptor signaling pathway	8/101	3.12E-06	3.87E-05	8
hsa04659	Th17 cell differentiation	8/101	6.52E-05	0.00054055	8
hsa04650	Natural killer cell mediated cytotoxicity	8/101	0.0002687	0.0017981	8
hsa04064	NF-kappa B signaling pathway	7/101	0.0002866	0.00184668	7

GO: Gene ontology; KEGG: Kyoto encyclopedia of genes and genomes.

### Identification of core genes for the most relevant immune infiltrates

**Figure 4:**
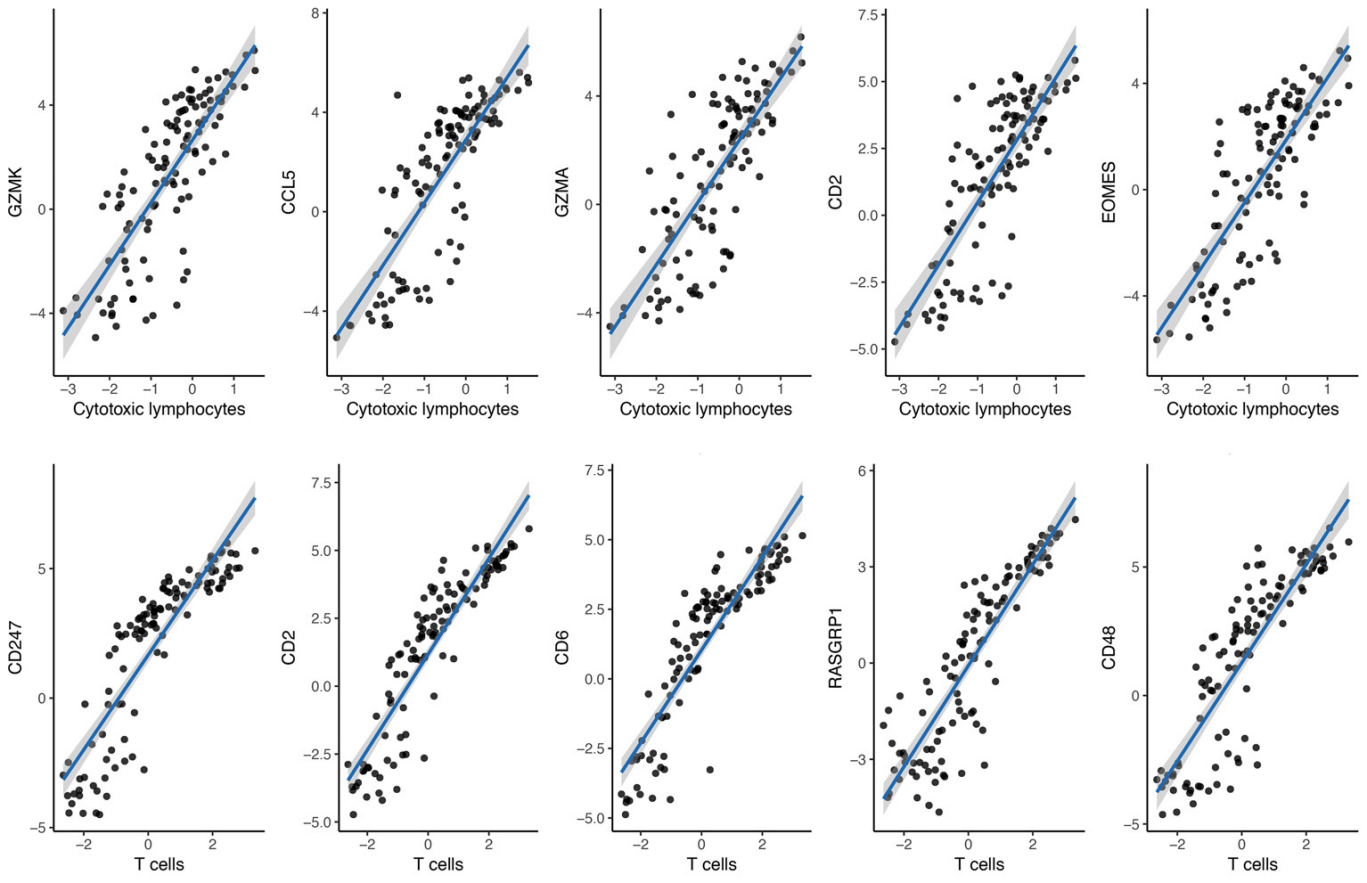
Correlation analysis of core genes with the most relevant immune infiltrates in abdominal aortic aneurysm (AAA).

We analyzed the relationship of genes in the red module with the infiltration of T cells and cytotoxic lymphocytes. We selected five genes with the strongest correlation as the core genes affecting immune infiltration in AAA. Cytotoxic lymphocytes were positively associated with the expression of granzyme K (GZMK), CCL5, granzyme A (GZMA), CD2, and eomesodermin (EOMES), while T cells showed significant correlations with CD247, CD2, CD6, RAS guanyl-releasing protein 1 (RASGRP1), and CD48 expression (Figure 4) (Table 2).

**Table 2:** Core gene correlation analysis results.

Type/Gene	r	P value	P_adjusted_
Cytotoxic lymphocytes			
*GZMK*	0.815	1.58E-28	3.24E-26
*CCL5*	0.812	3.28E-28	3.37E-26
*GZMA*	0.781	8.23E-25	5.62E-23
*CD2*	0.774	3.25E-24	1.67E-22
*EOMES*	0.770	7.80E-24	3.20E-22
T cells			
*CD247*	0.939	4.41E-54	9.05E-52
*CD2*	0.937	2.59E-53	2.65E-51
*CD6*	0.928	2.43E-50	1.66E-48
*RASGRP1*	0.905	8.57E-44	4.39E-42
*CD48*	0.900	1.82E-42	7.47E-41

## Discussion

Based on an integrative bioinformatics analysis, the current study took the first step to establish the patterns of immune infiltration in AAA using MCP algorithm. Six differential infiltrating cells were identified in AAA tissues. Then, we constructed gene co-expression network and conducted the functional and pathway enrichment analysis of genes in the most relevant module. Finally, we presented the immune-related core genes of T cells and cytotoxic lymphocytes. Our findings unveiled the inner link between profiles of immune infiltration and AAA risk, and rendered possible therapeutic targets for AAA.

Studies have explored immune infiltrating cells present in AAA tissues by IHC, which relied on a single surface marker to discern a subpopulation. However, this approach was considerably less efficient for discriminating closely relevant cell types, which could result in misleading and contradictory findings as many marker proteins were expressed in diverse cell types (15). Whether it is possible to break through a technical limitation to assess the overall immune infiltration and further analyze which subtype has a major effect in AAA formation is one emerging question.

In this study, we simultaneously quantified the relative proportions of ten subsets of infiltrating cells in AAA and normal tissues through MCP analysis, which was valid to compare the abundance of immune cells across multiple samples based on a rigorous set of markers (8,14). The results showed markedly elevated proportions of T cells, cytotoxic lymphocytes, NK cells, fibroblasts, myeloid dendritic cells, and neutrophils in AAA cases compared to normal aorta, indicating a promising role of these infiltrating subtypes in the risk of AAA. T cells, a heterogeneous group of lymphocytes with a diverse classification system and multiple physiologic actions, were observed to be a dominant population in AAA (16). The high prevalence of T cells in AAA patients suggested a critical role of adaptive immune cells in AAA pathophysiology (17). Cytotoxic lymphocytes were involved in the pathogenesis of AAA via the production of cytotoxic mediators, such as interferon-gamma and perforin, which resulted in the cytoskeletal destruction and smooth muscle cell (SMC) apoptosis (18,19). The balance of T cells and cytotoxic lymphocytes in acquired immunity is considered important in the process of aneurysm growth (20). Amin et al. (21) showed that T cell inhibitory molecule has the capacity to attenuate vascular inflammation and the content of several cell types including cytotoxic lymphocytes in aortic wall of AAA. Among innate immune cells in AAA patients, infiltrating NK cells can produce a high level of pro-inflammatory cytokines and perforin that might cause or exacerbate aortic tissue damage and increase the cytotoxicity against aortic SMCs (22). Dendritic cells are the most potent antigen-presenting cells that come in contact with T cells within lymphoid follicles and have a role in regulating the functional activity of immune response in AAA (23). Neutrophils appear to be one of the early contributors in AAA formation through secreting some specific ECM-degrading enzymes and neutrophil protease (3,24). Fibroblasts have also been implicated in AAA risk via influencing the secretion of inflammatory mediators and recruitment of immune cells to the aortic wall (25).

When correlation analysis of different modules with AAA-related immune infiltrates was performed, we found that the red module was strongly correlated with infiltrating levels of T cells and cytotoxic lymphocytes in the context of AAA. Further GO and KEGG analyses for genes in the most relevant module revealed a wide range of biological themes and pathways. Biological processes were particularly related to T cell activation, regulation of lymphocyte activation, leukocyte cell-cell adhesion, lymphocyte and mononuclear cell proliferation, and positive regulation of cytokine production. In addition, some pathways were associated with cytokine-cytokine receptor interaction, chemokine signaling pathway, T cell differentiation, and NF-kappa B signaling pathway. T cell activation and proliferation should be very central in the regulation of immune reactions in AAA (26). Cytokines in the aortic wall mainly secreted by T cells and cytotoxic lymphocytes could drive the aggregation of lymphocytes and their differentiation towards some effective phenotypes, further facilitating the adaptive immune response in AAA wall (27,28). Moreover, the trigger for immune cell recruitment may incorporate the elevated local production of chemokines, such as IL-8 and MCP-1 (29). The activation of NF-kappa B signaling pathway has been demonstrated to increase the expression and release of pro-inflammatory cytokines as well as proteases in AAA (6).

The activation of immune cells has been linked to the dynamic changes in gene expression, and related gene expression products can promote the inflammatory reactions in cells and tissues (30). We observed that enriched cluster of cytotoxic lymphocytes was characterized by a high expression level of GZMK, CCL5, GZMA, CD2, and EOMES genes, and there was a significant association of T cell infiltration with CD247, CD2, CD6, RASGRP1, and CD48 expression. GZMK and GZMA are members of the serine protease family detected in the granules of cytotoxic lymphocytes and have the ability to induce target cell apoptosis (31). Chemokine CCL5 is a target gene of NF-kappa B and plays an active part in recruiting a variety of leukocytes into inflammatory sites (32). As a T-box transcription factor, EOMES is expressed by activated cytotoxic cells and regulates the development and differentiation of certain effector cells (18). CD2 is a transmembrane glycoprotein located on T lymphocytes and cytotoxic cells with essential roles in immune recognition (33). CD247 is part of the T cell receptor complex and CD6 serves as a cell surface antigen, and both of them participate in the regulation of signal transduction in T cells (34,35). CD48 is a costimulatory receptor and contributes to T cell activation and proliferation through its interaction with CD2 (36). RASGRP1 is a guanine-nucleotide exchange factor known to control key immune cell functions, and its reductions are associated with immunodeficiency and even life-threatening immune dysregulation (37). Although the above factors have been found to exert immune regulatory effects in many diseases, their implications in AAA have never been defined. Our observations were indicative of the potential importance of these molecules in regulating the activation and differentiation of cytotoxic lymphocytes or T cells in AAA disease.

There were several limitations in our study. First, although we eliminated the imbalance of different microarray datasets by batch effect, heterogeneity of data in the public domain still existed in some level. Second, public datasets for analyzing gene expression profiles were limited, especially for normal abdominal tissues. Third, there was a lack of AAA-associated clinical information and current findings only relied on bioinformatics analysis. Thus, further *in vitro* and *in vivo* experiments are required to confirm our results and clarify the detailed mechanism.

In summary, the present study reflected a higher infiltration of T cells, cytotoxic lymphocytes, NK cells, fibroblasts, myeloid dendritic cells, and neutrophils in AAA. Functional annotation of genes in the most relevant module were carried out through GO and KEGG enrichment analysis. Furthermore, we identified marker genes strongly associated with the immune infiltration of cytotoxic lymphocytes and T cells. Our data may be beneficial to direct future research and the development of risk stratification and therapeutic options for AAA patients.

## Supplementary Material

Click here to view [pdf].
